# Dendritic Cells Release HLA-B-Associated Transcript-3 Positive Exosomes to Regulate Natural Killer Function

**DOI:** 10.1371/journal.pone.0003377

**Published:** 2008-10-13

**Authors:** Venkateswara Rao Simhadri, Katrin S. Reiners, Hinrich P. Hansen, Daniela Topolar, Vijaya Lakshmi Simhadri, Klaus Nohroudi, Thomas A. Kufer, Andreas Engert, Elke Pogge von Strandmann

**Affiliations:** 1 Laboratory of Immune Therapy, Department of Internal Medicine I, Centre for Integrated Oncology Koeln Bonn, University of Cologne, Cologne, Germany; 2 Institute for Anatomy-I, University Hospital of Cologne, Cologne, Germany; 3 Institute of Medical Microbiology, Immunology and Hygiene, University of Cologne, Cologne, Germany; Centre de Recherche Public-Santé, Luxembourg

## Abstract

NKp30, a natural cytotoxicity receptor expressed on NK cells is critically involved in direct cytotoxicity against various tumor cells and directs both maturation and selective killing of dendritic cells. Recently the intracellular protein BAT3, which is involved in DNA damage induced apoptosis, was identified as a ligand for NKp30. However, the mechanisms underlying the exposure of the intracellular ligand BAT3 to surface NKp30 and its role in NK-DC cross talk remained elusive. Electron microscopy and flow cytometry demonstrate that exosomes released from 293T cells and iDCs express BAT3 on the surface and are recognized by NKp30-Ig. Overexpression and depletion of BAT3 in 293T cells directly correlates with the exosomal expression level and the activation of NK cell-mediated cytokine release. Furthermore, the NKp30-mediated NK/DC cross talk resulting either in iDC killing or maturation was BAT3-dependent. Taken together this puts forward a new model for the activation of NK cells through intracellular signals that are released via exosomes from accessory cells. The manipulation of the exosomal regulation may offer a novel strategy to induce tumor immunity or inhibit autoimmune diseases caused by NK cell-activation.

## Introduction

NK) cells are lymphocytes involved in innate and adaptive immunity. Unlike T and B lymphocytes, NK cells do not possess antigen specific receptors. NK cell recognition of target cells and communication with different immune modulatory cells are regulated by a set of integrated signals provided by corresponding inhibitory and activating receptors [1], [2]. The well known killer-inhibitory receptors (KIRs) recognize the MHC-class molecules on self-cells and protect them against cytotoxicity. Virus-infected and tumor transformed cells down-regulate the MHC molecules and instead express inducible ligands for the activating receptor, NKG2D and the natural cytotoxicity receptors (NCRs) [3]. The knowledge on the ligands for NCRs is still limited. It has been shown that NKp30 and NKp46 recognize heparan-sulphate structures on tumor or virus-infected target cells [4]–[6]. We and others identified proteins residing within the target cells as potential NCR ligands including the intermediate filament-protein vimentin as an NKp46-binding protein [7] and the nuclear factor BAT3 as an NKp30-interacting partner [8]. However, it is still unclear how these intracellular proteins become exposed to the cell surface to activate NK cells. The contribution of such intracellular factors to the NK/DC cross talk is also not yet analyzed. This is of special interest for NKp30 and its tumor associated ligand BAT3, since NKp30 (NCR3, CD337) plays a crucial role in target-cell lysis as well as in the cross talk between NK cells and iDCs [9], [10].

Intracellular BAT3 has been reported to regulate apoptosis in a variety of settings [11]–[13]. Recently, p53 and apoptosis-inducing factor have been identified as BAT3 targets after DNA damage[14] and ER stress [15] respectively. Since extracellular BAT3 acts as a ligand for a natural killer cell receptor [8], it might constitute a molecular link from damaged cells to the immune system.

BAT3 contains a so-called BAG-domain (Bcl-2-associated athanogene domain found in the BAG family of proteins) that interacts with Hsp70 [12]. Reminiscent to BAT3, extra-cellular Hsp70 stimulates NK cell function and it is also involved in the activation of professional antigen-presenting cells such as macrophages and dendritic cells. Recently it was shown that intracellular Hsp70 is released via exosomes from tumor cells [16]. Exosomes are membrane micro-vesicles (diameter of 50–100 nm) [17] with the ability to direct the communication among cells in the immune system. Electron microscopy studies suggest that exosomes originate from the endocytic pathway called multi-vesicular bodies (MVBs) [23]. It is known that iDC-derived exosomes directly activate T cells in tumor immunity [18]. Moreover, pulsing of dendritic cells with immunogenic tumor-derived exosomes leads to T lymphocyte activation [19], [20].

Here we investigate the role of BAT3 for the NK-DC cross talk and the mechanisms that render the intracellular protein BAT3 accessible to the NKp30 cell-surface receptor.

## Results and Discussion

**Figure 1:**
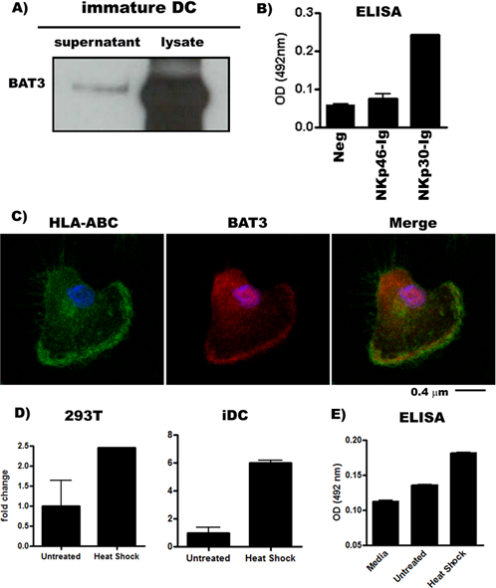
Expression of BAT3 on immature dendritic cells. (A) Western blot to detect BAT3 in total lysates and supernatant of heat shock treated monocyte-derived iDCs. (B). ELISA plates were coated with recombinant proteins, buffer control (Neg), NKp46-Ig and NKp30-Ig (concentration of 100 ng/ml), followed by incubation with 100 µl of concentrated supernatant obtained from heat shock treated iDCs and detected with anti-BAT3. Data represents absorbance at 492 nm. (C) Laser Scanning Microscopy to visualize HLA- A, B, C and BAT3 on dendritic cells upon staining with specific primary antibodies and labelled secondary antibodies. HLA-A, B, and C (green), BAT3 (red) and merge (right-yellow). Blue represents Hoechst33342 staining of cell nuclei. (D) Quantitative Real time PCR to detect BAT3 mRNA in 293T and iDCs upon exposure to heat shock. The Y-axis determines the fold change; where the untreated samples were normalized to factor 1. (E) Supernatant was collected from iDCs cells either untreated or treated with non-lethal heat shock (Heat shock) and analysed in specific BAT3 ELISA (sandwich method) to determine the amount of BAT3 in the supernatant. Error bars represent the standard deviation of duplicate samples. One representative experiment of three is shown.

BAT3 is a multifunctional protein that is released from tumor cells in response to cellular stress engaging NKp30 on NK cells [8]. Intracellular BAT3 is involved in apoptosis [13] and also in the p53-mediated gene transcription in response to DNA damage [14]. So far, nothing is known about the expression and function of BAT3 in dendritic cells. Since previous studies indicate that NKp30 plays a crucial role in triggering NK cell-mediated cytotoxicity and induces maturation of iDCs by engaging undefined ligands [9], [10], we initially analyzed the expression pattern of BAT3 in iDCs. BAT3 was detectable in lysates of monocyte-derived iDCs, and released into the extracellular compartment in response to non-lethal heat shock (Fig. 1A). Moreover, a BAT3- specific ELISA demonstrated that extracellular BAT3 derived from iDCS was specifically recognized by NKp30-Ig, whereas no binding to NKp46-Ig was observed (Fig. 1B). These data show that iDCs secrete BAT3, that specifically binds to NKp30 and support previous reports suggesting a role for NKp30 but not for NKp46 in the NK-DC cross-talk [9]. Laser Scanner Microscopy revealed that intracellular BAT3 was predominantly detectable in the cell nuclei and also on the cell membrane of heat shocked iDCs (Fig. 1C, merge). Interestingly, co-localization with surface MHC class I molecules (HLA-A, B, and C) was observed (Fig. 1C). Taken together, the expression profile of BAT3 in iDCs is in line with a potential NKp30 ligand.

The putative BAT3 promoter is glycine and cysteine rich and possesses heat shock elements at position -125 [21] and within the first intron of the ubiquitin-like domain of BAT3. Therefore we investigated whether BAT3 expression was regulated on the transcriptional level. Real-time PCR revealed an increase of BAT3 mRNA in response to a non-lethal heat shock in tumor cells and iDCs (Fig. 1D). The enhanced mRNA expression in dendritic cells was reproducible, although the induction varied from donor to donor. A consistent correlation of the BAT3 protein release estimated in a BAT3-specific sandwich ELISA (Fig. 1E) and the mRNA increase was demonstrated.

**Figure 2:**
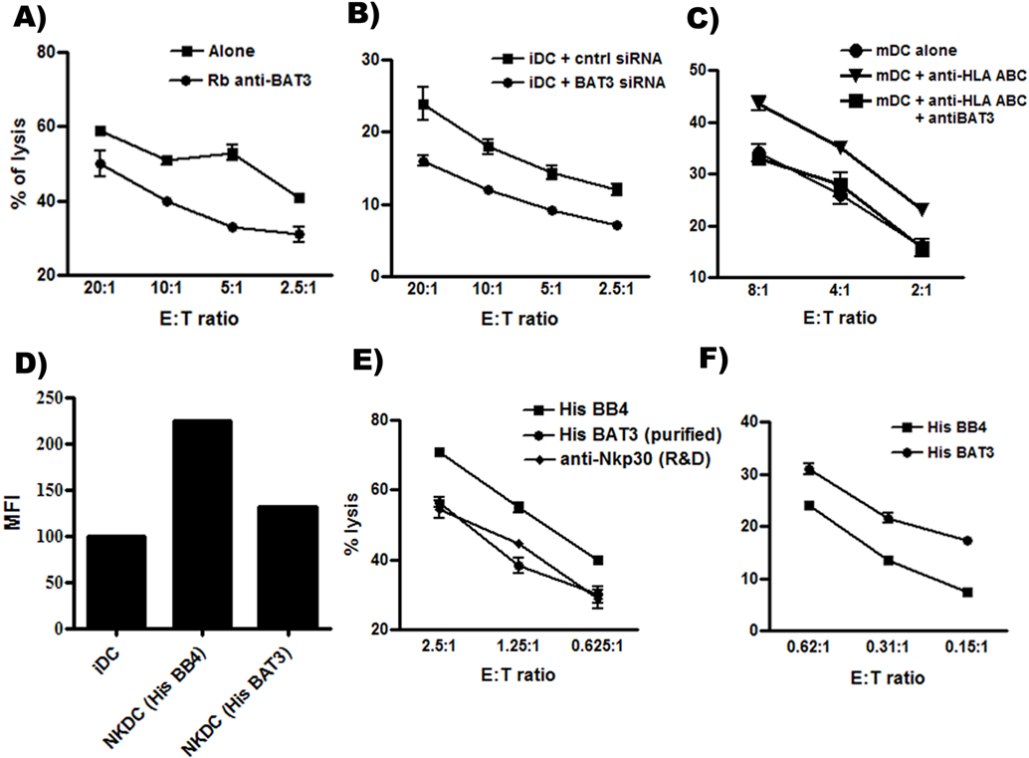
Role of BAT3 for iDCs and the effect of purified BAT3 on NK cell function. (A) Standard Europium release assay: Inhibition of NK-dependent lysis of iDCs in the presence of anti-BAT3 was significant (paired t-test, p-value = 0.008). (B) iDCs transfected with either control siRNA or BAT3 siRNA were co-incubated with NK cells for 4 hours at 37°C. The decrease of iDC lysis upon BAT3 down regulation was significant (p = 0.01). (C) Lysis of mature DCs upon pre-incubation with control antibodies, anti-HLA-ABC and/or anti-BAT3. (D) Co-culture of iDCs with activated NK cells at 5∶1 ratio (iDC∶NK) promotes the maturation of iDCs as shown by FACS analysis to detect expression of the maturation marker CD86. Inhibition of this effect is achieved by soluble purified BAT3. The y-axis represents the mean fluorescence intensity (MFI). (E) The lysis of Raji cells is inhibited by soluble BAT3 and anti-NKp30 compared to the control protein His BB4. The decrease of the lysis was significant (paired t-test, p value = 0.019). (F) NK cells were pre-stimulated with immobilized HisBB4 (control) and purified BAT3 prior a cytotoxicity assay with Raji cells as targets at different effector : target cell ratios. NK cells were derived from different donors for each experiment. Error bars for the lysis experiments represent standard deviation of three replicates. One representative experiment of four is shown.

We next analyzed the functional role of the NKp30-ligand BAT3 for iDC killing and maturation. A europium release assay with activated NK cells as effector cells and monocyte- derived iDCs as target cells was performed in the presence or absence of BAT3-masking antibodies. Addition of anti-BAT3 (rabbit polyclonal, [8]) inhibited NK cell mediated cytotoxicity significantly when compared to a rabbit control serum ((*p* = 0.019); Fig. 2A).

Moreover, down regulation of BAT3 protein upon nucleofection of BAT3 siRNA into iDCs resulted in a decrease of cytotoxicity compared to the control siRNA (Fig. 2B).

The maturation of DCs leads to high expression of MHC class-I molecules and thus protects from NK-mediated cytotoxicity. It is well known that mature DCs (mDCs) are less susceptible to lysis as compared to iDCs [9]. However, subsequent blocking of MHC-class-I molecules on mature DCs with HLA-A, B, C specific mAb lead to efficient lysis by NK cells and this effect could be blocked by adding BAT3 antibodies (Fig. 2C).

The NKp30-dependent NK-DC cross talk also results in iDC maturation (*12*). Thus, we explored the role of soluble protein on the maturation of iDCs in NK-DC-co-culture experiments and analyzed the expression of the co-stimulatory molecule CD86. As expected co-cultivation of monocyte-derived iDCs with NK cells induced enhanced expression of CD86 (Fig. 2D). In this setting the up-regulation of CD86 was reduced in the presence of soluble recombinant BAT3 (Fig. 2D) indicating that BAT3 has a substantial impact on the NK cell-mediated iDC-maturation.

The effect of recombinant BAT3 on NK cell cytotoxicity has not been addressed so far. Therefore we performed europium release assays using NK cells either pre-stimulated with soluble or with immobilized recombinant BAT3. We observed that NK-dependent lysis of Raji cells was inhibited when NK cells were stimulated with purified soluble HisBAT3 (Fig. 2E). Similar inhibition was achieved upon blocking the NKp30 receptor with a masking monoclonal antibody. The control protein (HisBB4) did not alter NK cell cytotoxicity. A similar reduction in NKp30-mediated cytotoxicity was also reported for the viral ligand pp65 [22]. On the other hand, we observed that immobilized BAT3 had an opposite effect enhancing the cytotoxicity of NK cells against the target cells compared to the control protein HisBB4 (Fig. 2F). This data confirm previous results indicating that recombinant BAT3 in a soluble form inhibits cytokine secretion of NK cells, whereas immobilized BAT3 activates TNF-α and IFN-γ release [8]. Taken together, it was demonstrated that the NKp30-mediated DC-killing and DC maturation is at least partly dependent on BAT3.

To assess the underlying mechanisms of BAT3 on NK cell function as an inhibitor in soluble form and an activator when immobilized, we directly tested whether BAT3 is secreted in a complex structure such as exosomes. Exosomes are membrane microvesicles (diameter of 50–100 nm) [17] that direct the communication among cells in the immune system. Tumor cells secrete exosomes similar to immune regulatory cells such as antigen-presenting cells (APC), T cells, reticulocytes and platelets.

**Figure 3:**
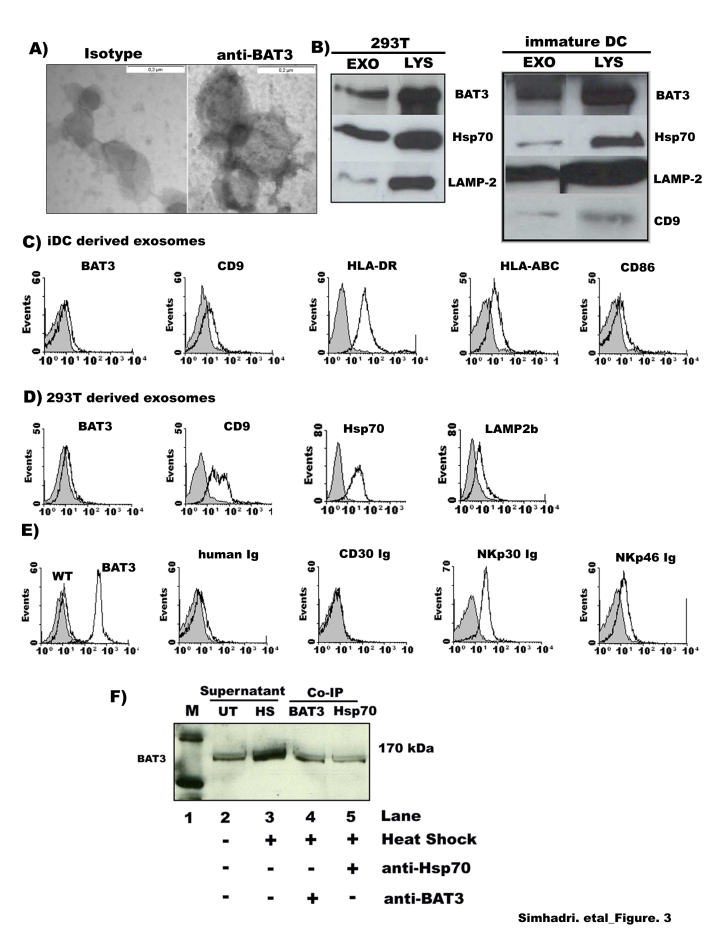
Bio-chemical characterization of the released BAT3. (A) Detection of BAT3 expression on exosomes by electron microscopy {left panel: gold antibody control (140000×) and right panel: exosomes stained with anti-BAT3 antibody (140000×)}. (B) Western blotting to detect BAT3, Hsp70, Lamp-2 and CD9 in exosomal fractions (30 µg) and lysate (10 µg) of 293T cells and iDCs. (C,D) FACS analysis to detect BAT3 and various surface markers on exosomes, that were purified from iDCs (C) or 293T cells (D) that were immobilized to latex beads. Grey background represents isotype control. (E) FACS analysis of exosomes derived from control transfected (wt) or BAT3-transfected (BAT3) 293T cells revealed over-expression of BAT3 on the exosomal surface. Specific binding of anti-BAT3, NKp30-Ig and NKp46-Ig was detectable. Grey histograms: background (secondary antibody) staining of beads coated with exosomes. (F) Western blot analysis demonstrates that the enhanced secretion of BAT3 into the supernatant obtained from tumor cells (293T) when treated with heat shock (HS, lane: 3) or left untreated (UT, lane: 2). Lanes 4 and 5 demonstrate the co-immunoprecipitation of BAT3 by using either a polyclonal BAT3 antibody (4^th^ lane) or a monoclonal Ab against Hsp70 (5^th^ lane). The western blot is stained for BAT3. Lane 1 (M) indicates the marker.

Exosomes were purified by ultracentrifugation from supernatant of 293T cells and their presence was confirmed by electron microscopy (Fig. 3A). As expected, the exosomes appeared as clusters of vesicles each surrounded by a double layer membrane. A specific immunostaining for BAT3 was detectable indicating that the exosomes expressed BAT3 (Fig 3A, right panel). This was confirmed by Western blot analysis of exosomes secreted from tumor cells and from iDCs (Fig. 3B). Moreover, sucrose gradient analysis was performed proving that BAT3 was associated with membrane vesicles (data not shown). The analysis of several control proteins revealed a differential expression for Hsp70, Lamp2 and CD9 between exosomes derived from tumor cells versus iDC-derived exosomes (Fig. 3B).

Until today little is known on the expression pattern of NCR ligands. Their cellular distribution has been detected only on a limited number of tumor cells [24], [25]. A comprehensive analysis showed NKp30L expression on normal and neoplastic cells by staining with NKp30-Ig fusion constructs [26]. More specifically, NKp30-Ig staining was observed in intra-cellular vesicular compartments very similar to early endosomal marker [26] which corresponds to the formation of exosomes. This supports our observation that the cellular ligand BAT3 is localized in endosomal vesicles and secreted in exosomal form. However, our data cannot rule out the existence of different isoforms of BAT3 that are released in exosomal or in soluble form. For instance, the release of soluble inhibitory forms could be mediated through shedding activity, as described for the ligands engaging the NKG2D receptor [27]. The inhibitory role of soluble recombinant BAT3 observed in our experiments might be due to a passive blocking or an active repression of NKp30-mediated signalling.

To assess directly the expression of BAT3 on the surface of exosomes, we used flow cytometric analysis. Purified exosomes from iDCs (Fig. 3C) and 293T cells (Fig. 3D) were incubated with latex beads of 4 µm in diameter and stained with antibodies directed against BAT3 [11] and several exosomal proteins. The expression of BAT3 and CD-9 (a tetra spin) was clearly detectable on the surface of both exosomal fractions. iDC-derived exosomes expressed also CD86, HLA class-I and HLA-DR as previously described. Positive staining for Hsp70 and Lamp-2 on 293T-derived exosomes revealed the purity of exosomes (Fig. 3D). Ligands for the NKG2D receptor were not detectable using NKG2D-Ig staining (not shown), suggesting that NKG2D engagement is independent of exosomes derived from 293T cells.

We then used BAT3-overexpressing 293T cells to directly prove the binding of NKp30 to BAT3 on the exosomal surface. As determined by flow-cytometry, exosomes derived from BAT3-transfected 293T cells express high levels of BAT3 on their surface (Fig. 3E). Purified exosomes were stained with Ig fusion proteins (human-Ig, CD30-Ig, NKp46-Ig and NKp30-Ig). The binding of NKp30-Ig to BAT3 on exosomes was specific and not observed for human-Ig and CD30-Ig (Fig. 3E). These experiments suggest that exosomal BAT3 acts as a membrane associated molecule which is presented to NKp30. Interestingly, recognition of so far unknown ligands for NKp46 was detectable using NKp46-Ig. A coordinated expression of ligands for different NCRs on exosomes may explain the cross-stimulation of NCRs in response to selective activation [2], [28]. Staining of iDC-derived exosomes with human Ig constructs was not feasible since Fc-receptors are present on exosomes derived from human dendritic cells.

Next we analyzed whether extracellular BAT3 was detectable in a physical association with Hsp70. The interaction of BAT3 and Hsp70 was so far only analyzed with respect to the intracellular regulation of apoptosis [12], although it is known that Hsp70 is involved in the regulation of immune cells in its extracellular, exosomal form [16], [29]. Extracellular BAT3 was precipitated from tumor-cell-derived supernatant upon a non-lethal heat shock using antibodies either binding BAT3 or Hsp70 suggesting that there is a BAT3/Hsp70 complex (Fig. 3F). Thus, the association of BAT3 and Hsp70 suggests that both factors are associated with exosomes and may act in combination to activate NK cells. It is known that heat shock does not influence the exosomal secretory rate, but it significantly enhances the Hsp70 content of exosomes isolated from cell supernatant [30]. Since the exosomal BAT3-expression is also induced in response to heat shock, both factors might act as intracellular danger sensors in a coordinative manner.

**Figure 4:**
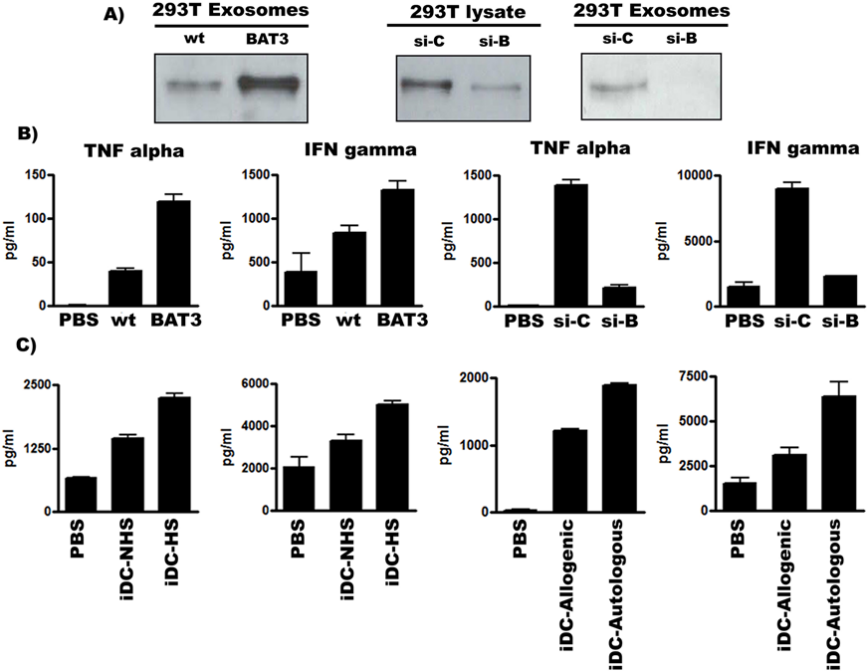
Exosomal BAT3 regulates NK cell-function. (A) Western Blot to detect BAT3 in exosomal fractions upon over expression and depletion. Exosomes purified from untransfected cells (WT), BAT3-transfected cells (BAT3), control siRNA- transfected cells (si-c) and BAT3 siRNA- transfected cells were analysed by Western blotting to detect BAT3. (B) Exosomes were purified from media (PBS), untransfected 293T cells (wt), BAT3-transfected 293T cells (BAT3), control si-RNA (si-c) and BAT3 si-RNA (si-B) transfected 293T cells and used to stimulate NK cells. NK cell-supernatant was collected and used for a cytokine ELISA (TNF-α and IFN-γ). (C) NK cells were stimulated with exosomes derived from untreated iDCs (iDC-NHS exosomes) or upon heat shock (iDC-HS exosomes) for cytokine ELISA (left panels). NK cell-mediated cytokine release was estimated upon stimulation with exosomes derived from allogenic and autologous iDCs (right panels). Primary immune cells were derived from different donors for each experiment. The means of duplicates and the concentration (pg/ml) are indicated. One representative experiment of three is shown.

We subsequently investigated the biological role of BAT3 surface-positive exosomes for NK cell activation. Exosomes with different BAT3 expressions levels were obtained from wildtype 293T cells (wt), upon BAT3-overexpression (BAT3) or siRNA-mediated down regulation (si-B) (Fig. 4A). NK cells were stimulated with these exosomes and the supernatant was collected for TNF-α and IFN-γ ELISA. Wild-type exosomes clearly stimulated the release of the inflammatory cytokines, and as expected, the cytokine release was even increased in response to BAT3- overexpressing exosomes (Fig. 4B). Interestingly, NK cells treated with BAT3-depleted exosomes failed to produce TNF-α and IFN-γ, whereas a robust release was observed with control exosomes (si-c) derived from control si-RNA transfected cells. Analogous results were obtained with iDC-derived exosomes (Fig. 4C). Exosomes were purified from untreated iDCs or from heat shock treated iDCs to increase the expression level of BAT3 in the supernatant. The release of TNF-α and IFN-γ was in fact stronger in response to heat shock treated exosomes and observed in both allogenic and autologous settings. Thus, the biological activity of exosomes correlated directly with the BAT3 expression level indicating that BAT3 is crucial for the exosomal-dependent activation of NK cells.

The data demonstrate a novel role for exosomes released from accessory cells that initiate the innate function of NK cells and may shape the adaptive immune response in a BAT3-dependent manner. It was shown that BAT3 acts as a dendritic cell- associated ligand providing an explanation how and when BAT3 - a intracellular protein - is secreted and engages the NKp30 receptor on effector cells. This is based on the following findings: (i) iDCs and tumor cells release BAT3 surface-positive exosomes; (ii) exosomes release was induced by moderate heat shock and correlated with an increase of BAT3 mRNA level; (iii) iDC-derived BAT3 positive exosomes induce cytokine secretion and (iv) BAT3 is critically involved in NK cell-dependent maturation and killing of iDCs. The association of extracellular BAT3 with exosomes suggests that BAT3 activates NK cells in combination with “co-factors” engaging receptors distinct from NKp30. This is further supported by the observation that BAT3-mediated NK cell activation cannot be completely blocked using masking NKp30 antibodies [8]. It is also known that several triggering NK receptors are activated in a coordinative manner [28]. Confirming this model it was shown recently that Hsp70 surface-positive exosomes and inducible NKG2D ligands expressed on tumor cells synergistically promote the activation of mouse NK cells resulting in a reduced in vivo tumor growth and progression [31].

One pathway that induces the expression of NKG2D ligands is the p53-mediated DNA damage response [32], [33]. It is known that BAT3 is crucial for the post-translational modification of p53 allowing the transcriptional up-regulation of p53-target genes such as p21 and puma [14]. The cellular DNA damage-response results also in the enhanced secretion of exosomes mediated via TSAP6 (tumor suppressor-activated pathway 6) that is a direct p53 transcriptional target gene [34], [35]. Further experiments will elucidate whether BAT3 is the key component linking the nuclear DNA damage response to the immune system via the release of immune-stimulatory exosomes.

The exosomal release of danger signals that alert NK cells may be considered as a priming signal and is in favour with a two-step activation model of human NK cells involving a priming and a triggering event. The NK cell activation via extracellular factors may thus result in yet unappreciated bystander effects exerted by NK cells. Recently, it was also demonstrated that resting NK cells acquire general functionality through *trans*-presentation of IL-15 by DCs [36]. A “priming” event has also been described for NK cells in tumor cell recognition, since the NK cell-mediated killing of initially resistant tumor cells was dependent on unknown extracellular factors derived from unrelated tumor cells that were sensitive for NK cell lysis [37].

Direct in vivo evidence for the interaction of BAT3/NKp30 for the recognition and elimination of tumor cells remains difficult since NKp30 is a pseudogene in mouse strains [38]. However aberrant expression of BAT3 is observed in certain human tumors such as malignant melanoma [39] and its identification as a putative tumor suppressor gene in colon carcinoma [40] clearly suggest a role in tumor biology.

## Materials and Methods

### Cells, cell transfection, proteins and antibodies

293T cells, a human fibroblast kidney cell line, were used for the purification of exosomes. Human NK cells were separated from peripheral blood mononuclear cells (PBMCs) using the NK Cell Isolation Kit and VarioMACS (Miltenyi, Bergisch Gladbach, Germany).

Separated polyclonal NK cells were cultivated in RPMI medium supplemented with 50 µg/mL penicillin, 50 µg/mL streptomycin, 10% fetal calf serum and 10 U/mL recombinant human IL-2 (R&D Systems, Wiesbaden, Germany) at 37°C with 5% CO_2_. Primary immature dendritic cells (iDCs) were differentiated from adherent peripheral blood monocytes in the presence of IL-4 and GM-CSF (Immunotools, Germany) at the final concentration of 20 ng/ml and 50 ng/ml, respectively for 5 days. For maturation of the DCs we have used TNF-α at a concentration of 50 ng/ml for 2 days. The purity of NK cells (CD3−, CD56+, NKp46+ and NKp30+) and DCs (CD14−, CD1a+, CD80+ and CD86+) was evaluated by flow-cytometric analysis. All the conjugated antibodies are from BD biosciences except NKp30 and NKp46 (Beckman Coulter).

The sequence encoding the extracellular domain of NKp30 was amplified by PCR from NKL-derived cDNA and cloned into the expression vector pCDNA3.1 (Invitrogen, Karlsruhe, Germany), which contained the human IgG1 kappa leader sequence (N-terminal) and the genomic DNA of the Fc portion of human IgG1 (C-terminal). The CD30 extracellular sequence was inserted in frame between the IgG1 kappa leader and the genomic DNA of the Fc portion of human IgG1. The control human IgG1 was obtained from pFuse2 vector. Human-Ig, NKp30-Ig and CD30-Ig fusion proteins were purified from the supernatant of transfected 293T cells with protein-G sepharose beads as recommended by the manufacturer (Amersham Bioscience, Freiburg, Germany).

Recombinant NKp46-Ig was purchased from R&D Systems, Wiesbaden, Germany.

DNA fragments encoding BAT3 full length were cloned into the expression vectors pCDNA3.1-His (Invitrogen, Karlsruhe, Germany) in frame with a C-terminal 6xhistidine tag (His)_6_. BAT3-transfected 293T cells were exposed to a non-lethal heat shock at 42°C for 30 minutes followed by a recovery period at 37°C for two hours. The supernatant was collected and purified using NiNTA affinity chromatography according to Qiagen, Hilden, Germany (The QIAexpressionist, 2003) dialysed against PBS.

We used the following primary antibodies: Anti-NKp30 (mAb1849, R&D Systems, Wiesbaden, Germany) for FACS and blocking experiments; BH1-2A as anti-NKp30 isotype control (kindly provided by Lemke, Kiel), anti-Hsp70 (WB-analysis-SPA810, Biomol, Hamburg, Germany), anti-LAMP2b (WB-analysis-ab18529, Abcam, Germany), anti-Hsp70 (FACS analysis- cmHsp70.1-kindly provided by G. Multhoff, München) and chicken polyclonal anti-BAT3 (ab37751, Abcam, Germany). The generation of the BAT3-specific rabbit antiserum (raised against the COOH-terminal region as an antigen ((NH2)-RKVKPQPPLSDAYLSGMPAK was described elsewhere[11]. The secondary antibodies were obtained from Dianova GmbH, Hamburg, Germany.

#### Immunoprecipitation

293T cells were transfected with an expression construct encoding histidine-tagged BAT3 using lipofectamine (Invitrogen, Karlsruhe, Germany). 48 hours post transfection the cells were treated with a non-lethal heat shock at 42°C for 30 minutes and recovered for 1–2 hrs at 37°C. The heat shock supernatant was collected and incubated with rabbit polyclonal sera against BAT3 and Hsp70 monoclonal antibodies (SPA810, Biomol, Hamburg, Germany) for one hour and followed by incubation with protein-A beads over night. The beads were washed and subjected to immunoblotting for detection of BAT3.

#### Isolation and Purification of Exosomes

Exosomes were purified as described elsewhere (Thery et al., 1999). In brief 293T cells were exposed to a non-lethal heat shock at 42°C for 30 minutes followed by a recovery period at 37°C for two hours (BAT3-SN). Exosomes were purified from the supernatant by three successive centrifugations at 300× g (5 min), 1200× g (20 min) and 10 000× g (30 min) to eliminate cells and debris, followed by centrifugation for 1 h at 100 000× g. The exosomal pellet was washed once in a large volume of PBS, centrifuged at 100 000× g for 1 h and re-suspended in PBS.

#### Electron Microscopy

PBS washed exosomal pellets were resuspended and fixed in 4% paraformaldehyde in PBS at 20°C for 15 minutes. Coated nickel grid was placed on top of a suspension drop followed by post-fixation with OsO4. The grids were immunolabeled with BAT3 specific antibodies followed by staining with 5 nm gold particles. Preparations were analyzed with a Zeiss EM-109 electron microscope (Carl Zeiss, Oberkochen, Germany).

#### ELISA binding assay of iDC supernatant to fusion proteins

Monocyte-derived immature dendritic cells were treated with heat shock for 30 minutes at 42°C and recovered at 37°C for another 30 minutes. The supernatant was collected and concentrated with viva-spin column (100 kDa cut-off) ensuring the concentration of higher-molecular weight structures. Different human-Ig fusion proteins (100 ng/well) were coated on plates in duplicates and were incubated with iDC supernatant derived from heat shock treated cells (the supernatant contains exosomes), for 2 hours at room temperature and detected using rabbit polyclonal BAT3 specific antibody and the corresponding anti-rabbit secondary antibody.

#### Coupling of exosomes and FACS Analysis of exosome-coated beads

Exosomes (30 µg) were incubated with 4.5-micron microsphere polybead carboxylate latex beads (Polysciences) for 30 minutes at room temperature. The beads were washed once with PBS and blocked with 2% BSA in PBS for 40 minutes. Beads were washed again for 2 times with PBS and incubated with various human Ig fusion proteins and different antibodies as specified, and analysed on a Becton Dickinson FACS Calibur using Cell Quest Pro software. Beads alone were gated and isotype-matched antibodies were used as controls for the fluorescence analysis.

#### mRNA Analysis

Total cellular RNA was isolated from heat shock-treated and untreated 293T or immature dendritic cells using the Qiagen RNeasy Mini Kit according to the manufacturer's recommendations. Reverse transcription was carried out using the QuantiTect Rev. Transcription Kit (Qiagen). Quantification of the mRNA encoding for BAT3 was performed using LightCycler technology (Roche Diagnostics). Quantitative PCR was performed in a total reaction volume of 20 µl using the QuantiTect SYBR Green PCR Kit. The following primers were used: BAT3-for: CTATTATCCAGCAGGACATTCAGAG; BAT3 rev: GCTAAGGATCATCAGCAAAGG and for the internal control housekeeping gene c-abl, for: CTTCATCCACAGAGATCTTGCTG and c-abl-rev: ATACTCCAAATGCCCAGACG. Housekeeping gene and target gene were quantified simultaneously in duplicates in one LightCycler run, together with the appropriate non-template controls. The difference in RNA quality and quantity between samples was normalized as given by the ratio of the copy number of the target gene and the copy number of c-abl.

#### Immunofluorescence

Monocyte-derived immature dendritic cells were stressed with heat shock and were plated overnight on poly-lysine coated cover slips. The cells were washed with PBS and fixed with 4% para-formaldehyde for 15 min at 25°C and also by ice-cold methanol for 10 min at −20°C. After the cells were washed with PBS, they were blocked with 10% bovine serum in PBS for 1 h at 25°C and further incubated with optimally diluted primary antibodies for overnight at 4°C. The cells were washed and the respective primary antibodies were detected using fluorescently labelled secondary antibodies, goat anti-mouse Alexa-flour 488 and goat anti-rabbit Alexa-flour635 (Molecular probes, Invitrogen) diluted in blocking buffer. The nuclei were stained with Hoechst 33342 (DNA staining). The cover slips were mounted onto slides with mounting medium (aqua poly/mount – Polysciences, Inc). The slides were examined with FLUOVIEW FV1000 laser scanning microscope with an objective lens UPLSAPO 60× W NA:1.20. The images were processed using software Image J and Adobe Photoshop.

#### Transfection of BAT3 siRNA

1.5×10^6^ cells (triplicates) were transfected with control siRNA (Alexa 488 conjugated siRNA) and a specific siRNA targeting BAT3 (Qiagen) using quantifect transfection reagent method (Qiagen). AMAXA nucleofection protocol was used for the transfection into immature dendritic cells.

#### Cytotoxicity assays

The cytotoxicity was estimated in a standard 4 hour europium release assay in a 96-well micro titer plate in a total volume of 200 µl with 5×10^3^ target cells and at different effector: target ratios. When mentioned, NK cells (effector cells) were incubated with 30% serum for 30 minutes at 4°C, washed and incubated with the monoclonal anti-NKp30 (R&D Systems, mAb1849) in the final concentration of 10 µg/ml for 1 hour at 4°C to block NKp30. NK cells were stimulated with immobilized or soluble recombinant BAT3 at a concentration of 1 µg/ml for 24–36 hours and then used as effector cells. The blocking anti-BAT3 serum (rabbit polyclonal) was used in a 1∶1000 dilution. The MHC class I molecules on mature dendritic cells (preincubated with human serum) were blocked using HLA-A, B, C antibody (Mouse IgG1κ Clone: G46-2.6, BD Biosciences). In all the experiments, the spontaneous release did not exceed 25% of the maximum release of the target cells.

#### IFN-γ and TNF-α assay

5×10^4^ primary NK cells were incubated for 48 hours with medium or exosomes derived from 293T cells either vector (mock) or BAT3-transfected (BAT3-SN) and with purified exosomes derived from dendritic cells. The NK cell-derived supernatant was analyzed (final concentration 1∶10 diluted) using IFN-γ and TNF-α -ELISA Detection Kits (R&D Systems, Wiesbaden Germany). The absorbance of the plates was measured using the ELISA-reader α-Quant (Bio-Tek, Bad Friedrichshall, Germany). Cyotkine release experiments were performed from NK cells purified from PBMCs of different donors. Various batches of NK cells were used for activation in different settings.

#### Statistical analysis

The results of the NK cell-activation assays are indicated as means±standard deviation. Significance was calculated with the GraphPad Prism software (San Diego, CA) using student paired t test. For preparation of the figures – softwares such as Adobe Photoshop, Image J and WinMDI were used.
